# Analysis of corneal wavefront aberrations and corneal densitometry in eyes with epithelial basement membrane dystrophy

**DOI:** 10.1007/s10792-025-03896-6

**Published:** 2026-01-07

**Authors:** Klemens Paul Kaiser, Jakob Wend, Myriam Böhm, Thomas Kohnen, Ingo Schmack

**Affiliations:** Department of Ophthalmology, Goethe-University, Theodor-Stern-Kai 7, 60590 Frankfurt Am Main, Germany

**Keywords:** Anterior basement membrane dystrophy, Cogan microcystic epithelial dystrophy, Epithelial basement membrane dystrophy, Map-dot-fingerprint dystrophy, Scheimpflug, Wavefront aberrations

## Abstract

**Purpose:**

To investigate wavefront aberrations, as well as corneal optical densitometry (COD), in eyes with epithelial basement membrane dystrophy (EBMD) and the influence on visual acuity.

**Methods:**

In this cross-sectional study, 70 eyes of 70 patients (mean age 55.9 ± 14.0 years) with the central cornea involving EBMD were compared to 50 healthy eyes of 50 patients (mean age 58.8 ± 14.1 years) serving as controls. Wavefront aberrations of the anterior corneal surface and the total cornea were measured with the Pentacam AXL (Oculus Optikgeräte GmbH, Wetzlar, Germany), and calculated for the 6 mm central corneal zone. In addition, the COD (corneal light backscatter measured in grey scale units) of the anterior 120 µm of the central 0–2 mm, 2–6 mm, and 6–10 mm of the cornea was evaluated. Corrected distance visual acuity (CDVA) was correlated with wavefront aberrations and COD using Spearman correlation analysis.

**Results:**

EBMD resulted in significant higher peak-to-valley (PTV; median: 15.0 [interquartile range: 9] µm), square root of the sum of the squared higher-order aberrations (RMS-HOA; 0.77 [0.52] µm), astigmatism (1.06 [1.04] µm), coma (0.41 [0.44] µm), and trefoil (0.28 [0.40] µm) (all *p* ≤ 0.01). A moderate correlation was found especially between CDVA and PTV as well as RMS-HOA. EBMD led to a statistically significant higher COD (*p* < 0.01) in the central corneal 6-mm and correlated moderately with CDVA outcomes.

**Conclusions:**

Our study revealed a significant correlation between elevated wavefront aberrations and backscattering in eyes affected by epithelial basement membrane dystrophy. While COD demonstrates potential for diagnostic purposes, additional studies are necessary to ascertain its specificity and distinguish EBMD from other ocular surface disorders.

## Introduction

Epithelial basement membrane dystrophy (EBMD) is primarily a degenerative, hereditary or post-traumatic condition of the corneal surface that typically occurs in adulthood [1]. Former eponyms for EBMD include anterior basement membrane dystrophy, map-dot-fingerprint dystrophy, and Cogan microcystic epithelial dystrophy [1]. Characteristic clinical findings of EBMD include curvy lines and irregular islands of thickened, greyish cloudy epithelium basement membrane (maps). Irregular, roundish-oval, putty-grey intraepithelial opacities called "dots", "fingerprint lines", and a subepithelial pebble-glass-like pattern (bleb pattern) are also common [1]. Patients may be asymptomatic or complain about blurry vision [2]. EMBD is estimated to affect 2–3% of the population [3]. It affects patients of all ages and both sexes, with the most common age group at presentation being between 25 and 75 years [3].

Despite the subtlety of the morphological changes, some patients complain about significant visual disturbances. Due to often non-specific symptomatology and similarity to other ocular surface diseases, such as dry eye syndrome, EBMD is often not properly recognized [4, 5]. The clinical relevance of EBMD has grown, especially in the context of modern refractive and cataract surgery, as it can lead to postoperative refractive surprises and suboptimal visual performance in eyes following diffractive intraocular lens (IOL) implantation [3, 6].

Objective assessment of EBMD-related visual impairment is limited. However, previous studies suggest that eyes with EBMD may exhibit increased higher-order aberrations (HOA), which can compromise visual acuity even in the absence of significant visual impairment [6]. One study demonstrated that analysis of epithelial wavefront aberrations and thickness variability provides highly sensitive and specific biomarkers for the diagnosis of EBMD [7]. HOA are more complex wavefront aberrations that cannot be corrected with spectacles and are increasingly recognized as a meaningful metric for assessing corneal optical quality [8, 9]. Wavefront aberrations are commonly represented in optical systems using Zernike polynomials, where each term corresponds to a specific type of aberration [10].

In addition to wavefront analysis, corneal densitometry has emerged as a valuable diagnostic tool to quantify corneal transparency by measuring backscattered light [11, 12]. With the advent of devices such as the Pentacam (Pentacam® AXL, Oculus Optikgeräte GmbH, Wetzlar, Germany), which is based on the Scheimpflug principle to obtain high-resolution, non-contact images of the entire anterior segment within a few seconds [13]. In addition to corneal pachymetry and tomography, it also includes densitometry—a measure of backlight scatter. Densitometry is already routinely used to measure corneal transparency after various corneal procedures [14, 15].

The aim of this study was to evaluate corneal wavefront aberrations and anterior corneal optical densitometry (COD) data in eyes with EMBD in comparison to non-diseased eyes with a clinical clear and transparent corneal stroma.

## Methods

This cross-section study included patients diagnosed with EBMD who presented to a single center (Department of Ophthalmology, Goethe-University, Frankfurt, Germany) between January 2022 and May 2024. The study involved human participants and was approved by the Ethics Committee of the Goethe-University Frankfurt (approval number: 2024-2024). The principles of the Declaration of Helsinki were followed. Due to the retrospective design of the study, no informed consent was required for the use of anonymized, non-personalized data.

### Inclusion and exclusion criteria

Patients with characteristic morphologic signs of EBMD (e.g., maps, dots, fingerprint lines, bleb patterns) and clinical symptoms like blurred vision or recurrent corneal erosions and complete aberrometry and densitometry measurements using the Pentacam (Oculus Optikgeräte GmbH, Wetzlar, Germany) were included. In patients with unilateral involvement, only the eye affected by EBMD was included in the analysis. If both eyes showed clinical findings of EBMD, one eye was selected for further analysis using simple randomization.

Eyes with previous history of corneal surgery, ocular trauma or other pathologies affecting the cornea (e.g., keratoconus, corneal scars, other corneal dystrophies or degenerations etc.), incomplete documentation as well as documented conditions significantly affecting visual acuity (e.g., age related macular degeneration, diabetic retinopathy, dens cataract) were excluded.

The control group comprised individuals with healthy eyes who did not have a history of EBMD, trauma, or ocular surgery and who attended our clinic for routine examinations or were interested in refractive procedures. Patients with any form of ocular pathology other than cataract were excluded from the control group. In particular, medical records were checked for evidence of dry eye, which was also ruled out.

### Examinations

All eyes included in this study were previously examined by a high-resolution rotating Scheimpflug camera system (Pentacam AXL or AXL Wave, Oculus Optikgeräte GmbH, Wetzlar, Germany). Measurements with insufficient quality specifications (other than "*OK*") were not considered. Beside standard tomography parameters (flat and steep keratometry and axis, central corneal thickness, and corneal astigmatism) peak-to-valley (PTV; difference between the highest positive and lowest negative point of the total wavefront data in µm), HOA, and corneal densitometry measurement (corneal light backscatter), expressed in grey scale units (GSU), ranges from 0 (fully transparent) to 100 (completely opaque) were obtained in all eyes [14, 16]. In addition, corrected distance visual acuity (CDVA) and objective refraction were measured in all eyes with the Topcon KR-800S Auto-Kerato-Refractometer (Topcon Medical Systems, Inc., Oakland, NJ, USA).

HOA of the anterior corneal surface and the total cornea were calculated for the central 6.00 mm zone. HOA values included only higher-order aberrations, with lower-order aberrations excluded from the calculation. The root mean square (RMS) values were automatically calculated by the Pentacam integrated software including the total wavefront aberrations (RMS total), and total amount of HOA (RMS-HOA) including 3rd to 6th order. The Zernike coefficients are represented as "C coefficients" (e.g. ${C}_{3}^{1}$ and ${C}_{3}^{-1}$), which differ in the trigonometric component. It is also possible to display the Zernike coefficients as "$|C|$ vectors". In this case, the two polynomials are combined and the length and angle are calculated as vectors. In this analysis, the Zernike polynomials from the 3rd to the 6th order were collected as $|C|$ vectors. For the correlation analysis, PTV, RMS-HOA, astigmatism $|{C}_{2}^{2}|$, Coma $|{C}_{3}^{1}|$, and Trefoil $|{C}_{3}^{3}|$ of the total cornea were used.

**Fig. 1:**
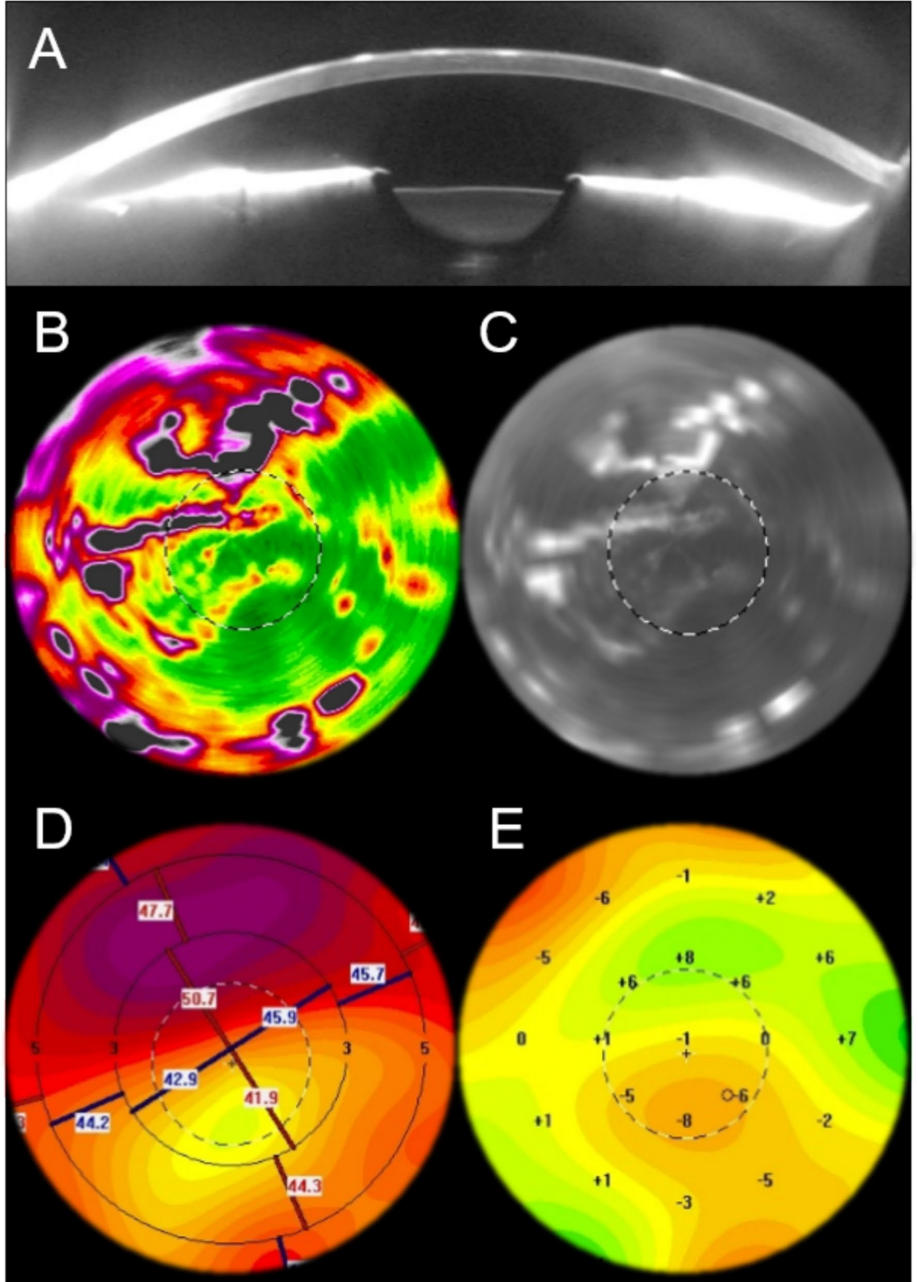
demonstrates characteristic signs in an eye with epithelial basement membrane dystrophy with hyperreflectivity of the anterior cornea in the sagittal Scheimpflug image (**A**). In corneal optical densitometry, maps, dots, and fingerprints can be seen in the Pentacam as a color representation (**B**) and as a grey value (**C**) of the central 6 mm of the cornea. The axial/sagittal curvature map of the anterior corneal surface shows an irregular surface (**D**). The elevation map of the anterior corneal surface indicates a thickening in the superior half of the pupil plane (**E**). In figures B-E, the black and white ring corresponds to the pupil

Corneal backscatter values were analyzed using the Cornea Densitometry Average Table, which is integrated in the Pentacam software, in four annular zones of the cornea centered at the corneal vertex. The present analysis included the first zone with a central diameter from 0–2 mm, the second zone from 2–6 mm, and the 6–10 mm zone. The 6–10 mm zone exerts a lesser influence on visual performance and quality; instead, it is more strongly influenced by other corneal changes, such as an arcus senilis. The analyses are provided for three layers, anterior corneal layer (ACL, first 120 μm), posterior layer (posterior 60 μm), and middle layer (between anterior and posterior layer without fixed thickness), as well as the total cornea [16]. Since EBMD represents a superficial corneal disorder, only the ACL was considered in this study. Figure 1 illustrates typical changes in an eye with EBMD in the Scheimpflug imaging using the Pentacam AXL (Oculus Optikgeräte GmbH, Wetzlar, Germany). In EBMD eyes where the affected corneal areas lacked sufficient documentation, particularly in regard to the involvement of the central cornea, the COD map was employed.

### Statistical analysis and sample size

Pseudonymized data were collected for all eyes and manually entered into an Excel spreadsheet (version 16.87; Microsoft Corporation, Redmond, WA, USA). Descriptive data were expressed in mean ± standard deviation (SD) if parametric, or median (interquartile range) if non parametric. Nominal or ordinal data were presented as totals and percentages. The Shapiro–Wilk test was used to determine normal distribution. The Wilcoxon signed-rank test (if nonparametric) or paired t-test (if normally distributed) was used to determine differences between eyes with clinical characteristic signs of EBMD and controls. Spearman correlation analysis was used to look for a potential correlation between wavefront aberrations, ACL COD, and CDVA. Furthermore, a multivariate linear regression analysis was performed to assess whether RMS-HOA remained independently associated with CDVA after adjusting for age and central corneal thickness (CCT). SPSS software (version 29.0.1.0; IBM Corporation, Armonk, NC, USA) was used for statistical analysis. Significance was defined as *p* < 0.05.

The post-hoc power analysis of the present study was based on the total corneas. The RMS-HOA mean value of 0.95 ± 0.53 was observed in the EBMD group (n = 70), while a mean value of 0.52 ± 0.24 was found in the control group (n = 50). The significance level (α) was set at 0.05. Test power was 0.9998.

## Results

### Patients’ demographics

A total of 70 eyes of 70 patients with characteristic clinical signs of EBMD were included in this study. Central corneal involvement in the pupillary plane was seen in all 70 eyes. The mean age was 55.94 ± 13.97 years (range: 25–81 years). The control group consisted of 50 healthy eyes of 50 patients with a mean age of 58.84 ± 14.08 years (range: 28–85 years). In the EBMD group, 41 patients (59.6%) were male and 29 (40.4%) females, while in the control group 27 patients (54.0%) were male and 23 (46.0%) females. In the EBMD group, 61 of the 70 eyes (87.1%) were phakic, while 9 eyes (12.9%) were pseudophakic. All control eyes were phakic. Demographic characteristics of both study groups are presented in Table 1.

**Table 1:** Patients demographic characteristics

Demographics	EBMD group (n = 70)	Control group (n = 50)	*p*-value
Age (years; mean ± SD, range)	55.94 ± 13.97; 25–81	58.84 ± 14.08; 28–85	0.293
Sex (female/male; n)	29/41	23/27	
Central corneal thickness (µm; mean ± SD, range)	557.60 ± 35.63; 454–631	545.90 ± 36.47; 480–665	0.158
Mean keratometry (D; mean ± SD, range)	43.63 ± 1.36; 40.2–46.8	42.98 ± 1.34; 40.0–46.4	0.001*
Corneal astigmatism (D; mean ± SD, range)	1.33 ± 0.84; 0.1–4.3	1.00 ± 0.74; 0.1–3.4	0.034*
Astigmatism with the rule (n [%])	35 (50.0%)	28 (56.0%)	
Astigmatism against the rule (n [%])	8 (11.4%)	7 (14.0%)	
Oblique astigmatism (n [%])	27 (38.6%)	15 (30.0%)	
Manifest refractive spherical equivalent (D; mean ± SD, range)	− 0.86 ± 3.10; − 7.38 to + 6.63	− 1.53 ± 3.64; − 10.75 to + 3.63	0.255
Visual acuity (logMAR; mean ± SD, range)	0.23 ± 0.49; 1.00 to − 0.08	− 0.03 ± 0.10; 0.30 to − 0.20	< 0.001*

Statistically significant differences are denoted by an asterisk (*)

*D* diopter, *EBMD* epithelial basement membrane dystrophy, *logMAR* logarithm of the minimal angle of resolution, *n* number, *SD* standard deviation

### Corneal wavefront aberration

As shown in Tables 2 and 3, there were significant differences between the EBMD group and the control group in regard to wavefront aberrations of the anterior corneal surface and the total cornea for the majority of parameters examined. There were also differences between the wavefront aberrations of the total cornea and the anterior corneal surface (Tables 2 and 3). Total corneas PTV was significantly higher (*p* < 0.01) with a median of 15.0 (8) µm in the EBMD group compared to a median of 8.0 (6) µm in the controls. The RMS-HOA of the total cornea showed a statistically significant higher value in the EBMD group with 0.77 (0.52) µm compared to the control group with 0.44 (0.27) µm (*p* < 0.01). Coma and trefoil of the total (both *p* < 0.01) also showed statistically significant differences. Table 2 gives a detailed overview of the total corneal wavefront aberrations and Table 3 of the wavefront aberrations of the anterior corneal surface of both groups. Table 4 illustrates the difference (Δ) between the wavefront aberrations of the total cornea and the anterior corneal surface.

**Table 2:** Total corneas wavefront aberrations

Parameters	EBMD group (n = 70)				Control group (n = 50)				*p*-value
	Median	IQR	Min	Max	Median	IQR	Min	Max	
Peak-to-valley (µm)	15.000	8.000	5.000	39.000	8.000	5.500	3.000	21.000	< 0.001*
RMS total (µm)	2.909	1.495	0.804	9.182	1.620	1.060	0.650	3.910	< 0.001*
RMS-LOA (µm)	2.744	1.496	0.732	9.008	1.570	1.050	0.580	3.870	< 0.001*
RMS-HOA (µm)	0.769	0.524	0.251	2.765	0.440	0.320	0.250	1.290	< 0.001*
Defocus $\left|{C}_{2}^{0}\right|$ (µm)	0.938	1.653	− 2.610	4.397	0.830	0.800	− 0.130	2.940	0.963
Astigmatism $|{C}_{2}^{2}|$ (µm)	1.061	1.043	0.037	4.360	0.743	0.713	0.072	3.352	0.005*
Coma $|{C}_{3}^{1}|$ (µm)	0.410	0.441	0.018	2.041	0.195	0.222	0.031	0.900	< 0.001*
Trefoil $|{C}_{3}^{3}|$ (µm)	0.281	0.401	0.038	2.223	0.143	0.166	0.010	0.536	< 0.001*
Spherical $|{C}_{4}^{0}|$ (µm)	0.253	0.253	− 0.305	0.841	0.269	0.185	0.025	0.622	0.515
Astigmatism 4th order $|{C}_{4}^{2}|$ (µm)	0.177	0.198	0.021	0.188	0.082	0.084	0.011	0.500	< 0.001*
4-Foil $|{C}_{4}^{4}|$ (µm)	0.191	0.206	0.022	0.888	0.105	0.089	0.012	0.444	< 0.001*
Coma 5th order $|{C}_{5}^{1}|$ (µm)	0.097	0.111	0.014	0.598	0.047	0.067	0.012	0.338	< 0.001*
Trefoil 5th order $|{C}_{5}^{3}|$ (µm)	0.068	0.086	0.003	0.522	0.035	0.028	0.004	0.290	< 0.001*
5-Foil $|{C}_{5}^{5}|$ (µm)	0.132	0.134	0.009	0.643	0.059	0.065	0.002	0.251	< 0.001*
Spherical 6th order $|{C}_{6}^{0}|$ (µm)	0.006	0.072	− 0.281	0.144	0.007	0.021	− 0.074	0.040	0.719
Astigmatism 6th order $|{C}_{6}^{2}|$ (µm)	0.047	0.054	0.002	0.310	0.023	0.024	0.002	0.151	< 0.001*
4-Foil 6th order $|{C}_{6}^{4}|$ (µm)	0.049	0.050	0.004	0.332	0.033	0.035	0.003	0.173	0.003*
6-Foil $|{C}_{6}^{6}|$ (µm)	0.066	0.105	0.000	0.437	0.035	0.035	0.006	0.153	< 0.001*

Statistically significant differences are denoted by an asterisk (*)

*EBMD* epithelial basement membrane dystrophy, *HOA* higher-order aberrations, *IQR* interquartile range, *LOA* lower-order aberrations, *Max* maximum, *Min* minimum, *logMAR* logarithm of the minimal angle of resolution, *n* number, *SD* standard deviation

**Table 3:** Corneal anterior surface wavefront aberrations and anterior corneal layer optical densitometry (ACL COD)

Parameters	EBMD group (n = 70)				Control group (n = 50)				*p*-value
	Median	IQR	Min	Max	Median	IQR	Min	Max	
Peak-to-valley (µm)	15.500	8.000	5.000	44.000	10.000	5.000	5.000	22.000	< 0.001*
RMS total (µm)	3.075	1.541	0.775	9.192	1.915	0.908	0.989	4.414	< 0.001*
RMS-LOA (µm)	2.944	1.493	0.551	9.022	1.786	0.928	0.914	4.378	< 0.001*
RMS-HOA (µm)	0.768	0.558	0.297	3.048	0.444	0.270	0.263	1.196	< 0.001*
Defocus $\left|{C}_{2}^{0}\right|$ (µm)	1.172	1.528	− 2.494	6.696	1.057	0.689	− 0.014	2.061	0.721
Astigmatism $|{C}_{2}^{2}|$ (µm)	1.190	1.009	0.172	4.343	0.927	0.884	0.169	3.846	0.017*
Coma $|{C}_{3}^{1}|$ (µm)	0.441	0.454	0.008	2.270	0.200	0.222	0.095	0.791	< 0.001*
Trefoil $|{C}_{3}^{3}|$ (µm)	0.316	0.391	0.033	2.198	0.139	0.170	0.009	0.525	< 0.001*
Spherical $|{C}_{4}^{0}|$ (µm)	0.281	0.264	− 0.305	0.924	0.287	0.168	0.049	0.626	0.647
Astigmatism 4th order $|{C}_{4}^{2}|$ (µm)	0.166	0.197	0.014	0.801	0.071	0.064	0.008	0.547	< 0.001*
4-Foil $|{C}_{4}^{4}|$ (µm)	0.199	0.161	0.009	0.889	0.097	0.084	0.010	0.384	< 0.001*
Coma 5th order $|{C}_{5}^{1}|$ (µm)	0.091	0.099	0.007	0.613	0.048	0.060	0.007	0.342	< 0.001*
Trefoil 5th order $|{C}_{5}^{3}|$ (µm)	0.071	0.070	0.008	0.483	0.036	0.029	0.001	0.187	< 0.001*
5-Foil $|{C}_{5}^{5}|$ (µm)	0.128	0.151	0.010	0.741	0.054	0.064	0.010	0.253	< 0.001*
Spherical 6th order $|{C}_{6}^{0}|$ (µm)	− 0.009	0.068	− 0.290	0.138	− 0.002	0.022	− 0.077	0.032	0.991
Astigmatism 6th order $|{C}_{6}^{2}|$ (µm)	0.048	0.064	0.001	0.297	0.022	0.024	0.000	0.147	< 0.001*
4-Foil 6th order $|{C}_{6}^{4}|$ (µm)	0.051	0.052	0.006	0.346	0.031	0.035	0.004	0.420	0.002*
6-Foil $|{C}_{6}^{6}|$ (µm)	0.062	0.109	0.004	0.464	0.031	0.044	0.003	0.123	< 0.001*
*Corneal optical densitometry*									
ACL COD 0–2 mm (GSU)	30.800	6.075	23.500	55.800	27.200	3.100	22.500	32.700	< 0.001*
ACL COD 2–6 mm (GSU)	29.300	6.075	20.900	47.400	25.900	5.200	20.000	36.800	< 0.001*
ACL COD 6–10 mm (GSU)	35.600	15.475	17.200	68.700	35.300	6.200	22.200	72.600	0.951

Statistically significant differences are denoted by an asterisk (*)

*EBMD* epithelial basement membrane dystrophy, *GSU* gray scale units, *HOA* higher-order aberrations, *IQR* interquartile range, *LOA* lower-order aberraitons, *Max* maximum, *Min* minimum, *logMAR* logarithm of the minimal angle of resolution, *n* number, *SD* standard deviation

**Table 4:** Difference (Δ) in wavefront aberrations between the corneal anterior surface and the total cornea

Parameters	EBMD group (n = 70)				Control group (n = 50)				*p*-value
	Median	IQR	Min	Max	Median	IQR	Min	Max	
ΔPeak-to-valley (µm)	− 1.000	2.000	− 6.000	3.000	− 1.000	2.000	− 4.000	2.000	0.930
ΔRMS total (µm)	− 0.160	0.311	− 1.198	0.441	− 0.238	0.250	− 0.627	0.328	0.329
ΔRMS-LOA (µm)	− 0.171	0.309	− 1.160	0.475	− 0.244	0.245	− 0.636	0.331	0.363
ΔRMS-HOA (µm)	− 0.012	0.063	− 0.302	0.204	− 0.011	0.042	− 0.096	0.099	0.850
ΔDefocus $\left|{C}_{2}^{0}\right|$ (µm)	− 0.226	0.223	− 0.901	0.124	− 0.228	0.177	− 1.182	1.925	0.331
ΔAstigmatism $|{C}_{2}^{2}|$ (µm)	− 0.184	0.278	− 0.610	0.417	− 0.167	0.266	− 1.868	0.228	0.780
ΔComa $|{C}_{3}^{1}|$ (µm)	− 0.006	0.060	− 0.484	0.182	− 0.004	0.069	− 1.121	0.110	0.651
ΔTrefoil $|{C}_{3}^{3}|$ (µm)	0.003	0.106	− 0.490	0.209	0.001	0.061	-0.074	0.069	0.848
ΔSpherical $|{C}_{4}^{0}|$ (µm)	− 0.033	0.043	− 0.136	0.071	− 0.028	0.032	− 0.083	0.036	0.256
ΔAstigmatism 4th order $|{C}_{4}^{2}|$ (µm)	− 0.001	0.039	− 0.097	1.728	0.013	0.029	− 0.047	0.103	0.162
Δ4-Foil $|{C}_{4}^{4}|$ (µm)	0.008	0.041	− 0.205	0.101	0.007	0.045	− 0.067	0.098	0.491
ΔComa 5th order $|{C}_{5}^{1}|$ (µm)	0.001	0.021	− 0.053	0.057	0.001	0.015	− 0.032	0.044	0.825
ΔTrefoil 5th order $|{C}_{5}^{3}|$ (µm)	0.005	0.018	− 0.116	0.044	0.000	0.013	− 0.020	0.244	0.290
Δ5-Foil $|{C}_{5}^{5}|$ (µm)	0.001	0.043	− 0.091	0.177	− 0.001	0.023	− 0.063	0.093	0.657
ΔSpherical 6th order $|{C}_{6}^{0}|$ (µm)	0.010	0.010	− 0.054	0.049	0.010	0.009	0.000	0.020	0.298
ΔAstigmatism 6th order $|{C}_{6}^{2}|$ (µm)	− 0.001	0.016	− 0.033	0.022	0.001	0.012	− 0.018	0.027	0.360
Δ4-Foil 6th order $|{C}_{6}^{4}|$ (µm)	0.000	0.013	− 0.031	0.027	0.003	0.011	− 0.392	0.017	0.325
Δ6-Foil $|{C}_{6}^{6}|$ (µm)	0.005	0.017	− 0.034	0.099	0.000	0.013	− 0.027	0.057	0.183

*EBMD* epithelial basement membrane dystrophy, *GSU* gray scale units, *HOA* higher-order aberrations, *IQR* interquartile range, *LOA* lower-order aberrations, *Max* maximum, *Min* minimum, *logMAR* logarithm of the minimal angle of resolution, *n* number, *SD* standard deviation

### Corneal densitometry

The differences between the ACL COD of the central 0–2 mm and 2–6 mm were statistically significantly higher in the EBMD group compared to the control group (*p* < 0.01) and are shown in Table 3.

### Correlation between corneal wavefront aberrations and visual acuity as well as corneal densitometry and visual acuity

Correlations between wavefront aberrations and COD with CDVA, quantified by Spearman's correlation analysis, are summarized in Table 5. All examined parameters showed a negative correlation with CDVA for both groups. The highest negative correlation was found in the EBMD group for PTV (Rho = − 0.486, *p* < 0.01) and RMS-HOA (Rho = − 0.417, *p* < 0.01). Additionally, there was a moderately negative correlation between ACL COD and CDVA (0–2 mm; Rho: − 0.476, *p* < 0.01). In the control group, a moderate statistically significant negative correlation was found for RMS-HOA and ACL COD 2–6 mm (Rho = − 0.297, *p* = 0.04, and Rho = − 0.393, *p* < 0.01, respectively).

**Table 5:** Spearman’s correlation between wavefront aberrations, anterior corneal layer optical densitometry (ACL COD), and visual acuity

Parameters	EBMD group (n = 70)		Control group (n = 50)	
	Spearman-Rho	*p*-value	Spearman-Rho	*p*-value
*Wavefront aberrations*				
Peak-to-valley	− 0.486	< 0.001*	− 0.227	0.114
RMS-HOA	− 0.417	< 0.001*	− 0.297	0.036*
Astigmatism $|{C}_{2}^{2}|$	− 0.337	0.002*	− 0.034	0.814
Coma $|{C}_{3}^{1}|$	− 0.316	0.004*	− 0.017	0.907
Trefoil $|{C}_{3}^{3}|$	− 0.313	0.004*	− 0.240	0.093
*Corneal optical densitometry*				
ACL COD 0–2 mm	− 0.476	< 0.001*	− 0.256	0.072
ACL COD 2–6 mm	− 0.473	< 0.001*	− 0.393	0.005*
ACL COD 6–10 mm	− 0.391	< 0.001*	− 0.434	0.001*

Statistically significant differences are denoted by an asterisk (*)

*EBMD* epithelial basement membrane dystrophy, *HOA* higher-order aberrations

In a multivariate linear regression model with CDVA as the dependent variable, RMS-HOA, age, and central corneal thickness were entered as predictors. RMS-HOA remained an independent predictor of worse visual acuity (β = − 0.176, *p* < 0.01) after adjusting for age and CCT, and age also showed a significant negative association with CDVA (β = − 0.0075, *p* < 0.01), while CCT was not significant (*p* = 0.13). The model explained 31.5% of the variance in CDVA (R^2^ = 0.315).

## Discussion

In the era of modern and advanced cataract and refractive surgery, ocular surface disorders such as EBMD are becoming increasingly important and should not be neglected in the preoperative evaluation, as they may mimic false or induced astigmatism [3]. In clinical practice, EBMD often results in an irregular corneal surface with changes in the axis and amount of astigmatism over time, which may lead to postoperative refractive surprises [17, 18]. The corneal irregularity is primarily attributable to a thicker corneal epithelium, particularly in the central and inferior region, as evidenced by studies employing anterior segment optical coherence tomography [19–21]. Therefore, in the context of "refractive" cataract surgery with high patient’s expectations, the proper diagnosis and treatment of preexisting corneal disorders is often crucial [18]. Because EBMD may be misdiagnosed or confused with other ocular surface diseases (e.g. dry eye disease) imaging techniques are mandatory for an accurate identification of eyes with morphologic changes associated with EBMD [22]. Furthermore, it is necessary to understand how these changes impact visual quality and performance to guide surgical planning and patient counseling.

Bellucci et al. evaluated visual acuity and quality in 25 eyes (13 patients) with EBMD after cataract surgery with monofocal intraocular lens implantation and compared them with pseudophakic controls without EBMD [6]. Their findings illustrating significantly worse aberrometric parameters, as well as lower UCVA and DCVA in the EBMD group, align with our results. Mean CDVA was 0.18 ± 0.15 logMAR in EBMD eyes and 0.06 ± 0.04 logMAR in control eyes (*p* < 0.01), which is comparable to our findings of 0.23 ± 0.49 logMAR and − 0.03 ± 0.10 logMAR, respectively [6]. However, the study cohort of Bellucci et al. was on average almost ten years older, and the majority of eyes were pseudophakic, whereas most eyes in our study were still phakic [6]. Bellucci et al. employed the Optical Quality Analyzing System (OQAS II, HD Analyzer, Visiometrics SL, Terrassa, Spain), while our study used the Pentacam (Oculus Optikgeräte GmbH, Wetzlar, Germany) for measuring wavefront aberrations [6]. Since optical quality was assessed over a 4 mm zone in their study versus a 6 mm zone in ours as recommended by Thibos et al., direct comparison is not feasible [6, 23]. This variability in measurement zones raises important questions about standardization in corneal imaging. Larger diameters capture paracentral changes more relevant under scotopic conditions, while smaller diameters may reflect more central irregularities. Given that modern intraocular lens (IOL) calculation methods, particularly ray-tracing algorithms, rely on data from broader cornea zones, inconsistencies in measurement protocols could impact refractive outcomes in EBMD patients. However, the precise influence of EBMD-related corneal irregularities on IOL power calculation through ray-tracing remains to be elucidated.

Interestingly, in our cohort, mean central corneal thickness was slightly higher in EBMD patients compared to controls. This finding is in line with previous anterior segment OCT studies, which demonstrated epithelial thickening and irregular basement membrane deposition in EBMD, particularly in the central and inferior cornea [19, 21]. These structural changes may account for the modest pachymetric increase observed in our study, even though the difference did not reach statistical significance.

In our study, eyes with EBMD showed statistically significant higher values in nearly all wavefront aberration parameters compared to controls, except for defocus and primary and secondary spherical aberrations. Although defocus did not differ significantly between groups, this is expected because EBMD primarily affects HOA rather than spherical refractive components. This supports the concept that visual impairment in EBMD arises predominantly from irregular rather than regular refractive error. Especially 3rd-order HOA are particularly relevant for visual quality were notably elevated in the EBMD group. Correlation analysis revealed moderate negative correlations between visual acuity and PTV and RMS-HOA, as well as astigmatism, coma, and trefoil. While these findings underscore the relationship between specific higher-order aberrations and visual function, further clarification is mandatory to ascertain their clinical relevance. A fruitful avenue for future research would be to explore the potential of identifying dominant aberrations, such as coma or trefoil, to inform diagnostic strategies. The analysis of the anterior corneal surface showed a similar trend, supporting the anatomical localization of EBMD changes.

Corneal densitometry is an established method for objectively assessing corneal transparency [14, 16, 24]. Chaurasia et al. showed that eyes with EBMD and Fuchs endothelial corneal dystrophy showed statistically significantly higher densitometry values (40% vs. 27% controls) [24]. Our findings of significantly elevated anterior COD values in EBMD eyes are consistent with these results, though less pronounced as in the study by Chaurasia et al. likely due to the differences in study populations [24]. Importantly, our EBMD group included only eyes with EBMD and no other corneal pathologies. COD may therefore be a valuable diagnostic tool, especially when slit lamp findings are subtle.

The extent to which COD can be used to differentiate EBMD from other ocular surface diseases remains to be elucidated. As shown in Fig. 1, the Pentacam’s color-coded densitometry may reveal characteristic surface alterations even in the absence of the patient. While the characteristic corneal changes of EBMD can be effectively visualized and mapped using COD, facilitating its differentiation from other ocular surface conditions (e.g., dry eye disease, keratopathy, guttate cornea), it may be challenging to define a specific numerical threshold for clinical application. In light of the significant rise in the deployment of artificial intelligence and algorithms, COD could potentially help distinguish EBMD from other ocular surface diseases. Nevertheless, this requires further investigation and algorithm development.

Our study has several limitations. The retrospective nature of the study and the use of autorefractometry for visual acuity testing limit generalizability. To date, it remains unclear whether autorefractometer-based measurements yield results comparable to subjective refraction in EBMD eyes. The lack of repeatability of HOA in healthy eyes and the effects of EBMD on the repeatability of corneal measurements must be taken into account, whereby only one measurement was performed in each case in the present study. The phakic status in most EBMD eyes complicates interpretation of visual outcomes, and the exact etiology of EBMD (degenerative vs. traumatic) could not be definitively determined due to insufficient documentation. Prospective longitudinal studies are warranted to better understand how EBMD affects aberrations and densitometry in EBMD, as well as the diagnostic utility of HOA or COD.

In conclusion, the results of our study demonstrated a notable elevation in wavefront aberrations and backscatter in eyes affected by clinical characteristic signs of EBMD, which exhibit a correlation with visual acuity. While COD appears promising as a supportive diagnostic tool, further studies are required to explore its specificity, to define clinical thresholds, and to assess its role in distinguishing EBMD from other ocular surface disorders.

## Data Availability

All data generated or analyzed during this study are included in this article. Further enquiries can be directed to the corresponding author.
